# Supplementary material for: ‘But Because I Don’t Know About It, That’s Why I Haven’t Done It’: Experiences of Access to Preventive Sexual and Reproductive Health Care for Refugee Women from Iraq and Syria Living in Melbourne, Australia—A Qualitative Study
Source: Int J Environ Res Public Health. 2025 Jan 23;22(2):149. doi: 10.3390/ijerph22020149 (PMC11854993; doi:10.3390/ijerph22020149)
Supplement: Supplementary file 1 [file ijerph-22-00149-s001.zip › ijerph-3296796-supplementary.pdf]

Supplementary File S1 Interview Guide

**KNOWLEDGE AND UNDERSTANDING**<br>In Australia, people go to the doctor when they are sick. But people also go to the doctor or nurse when they are not sick to have tests to make sure they protect their health. For example, if they want to avoid pregnancy, protect their health and stop the infection that can cause cancer. In Australia we call that prevention.		
**Prevention-specific information, care and services**	Have you seen a doctor or nurse since arriving in Australia?<br><br>Have you seen a doctor or a nurse when you were not sick?<br><br>There are some health problems related to women's overall health and wellness that only women experience.	
**Level of knowledge and understanding (UNDERSTANDING) Knowledge gaps**	By women's health problems I mean using contraception, cervical screening or pap tests, breast screening and human papilloma virus injections?<br><br>Have you heard about women's health problems?<br><br>Have you been to see a doctor or nurse about these women's health problems?	*[If no]* Who do you speak to/ where do you go if you have questions about women's health?
**Contraception**	For example, have you heard about how to stop having babies or get help with birth spacing? In Australia this is called contraception. Do you have a word for it? Are you doing or using something to help you not have babies now?<br><br>*[If no]* In your opinion what would make you decide to use or not use contraception?<br><br>*[If yes]* Can you tell me how did you find out about these methods? And what it *(the experience with the doctor or nurse)* was like?	
**Cervical screening**	In Australia, women *[aged 25 to 74 years]* are encouraged to have a test, called a cervical screening or pap test, to make sure that problems are detected early.<br><br>Have you heard about the cervical screening test or pap test? Do you know why it's done?<br><br>Have you ever had cervical screening?<br><br>*[If yes]* Can you tell me what it *(the experience with the doctor or nurse)* was like?<br><br>*[If no]* In your opinion what would make you or women in your community have or not have cervical screening?	Are there things you find confusing or that you'd like to know more about?

**HPV vaccination**<br><br><br><br><br><br><br><br><br><br><br><br><br><br><br><br><br><br><br><br><br>**Breast screen**	Young people *[adolescents aged 9–18 years]* in Australia can now have a free injection or needle that helps reduce the risk of infection that leads to cervical cancer.<br><br>In Australia high schools give young people cards issued in English. The cards' title is "Human papillomavirus (HPV) Immunisation consent card" it is a about a needle that is recommended to all students aged 9 to 18 to help protect their health.<br><br>Have you heard about this injection? Do you know why it's suggested and why it's done?<br><br>Has your daughter or son been offered this injection *[and have you seen this card]?*<br><br>If it was offered, how would you feel about your daughter or son having this injection?<br><br>*[If no]* In your opinion what would make you decide to let your daughter or son have or not have a HPV injection?<br><br>In Australia, women over 50 are encouraged to have a test, called a breast screen, to make sure that problems are detected early.<br><br>Have you heard about breast screen or mammography from your mother or other women in your community? Do you know why it's done?<br><br>Have you ever had a breast screen?<br><br>*[If yes]* Can you tell me what it (*the experience with the nurse or radiographer*) was like?<br><br>Has your mother or other women in your community been offered a breast screen?<br><br>*[If no]* In your opinion what would make you, your mother or other women in your community decide to have or not have a breast screen?	Are there things you find confusing or that you'd like to know more about?<br><br><br><br><br><br><br><br><br><br><br><br>Are there things you find confusing or that you'd like to know more about?
Experiences of using services		
**(UNDERSTANDING, ACCESS, HEALTH SERVICE USE) Unmet health need (ACCESS) (HEALTH SERVICE USE and UNDERSTANDING)**	When you did see a doctor or a nurse….<br><br>Were there any problems? How were you treated? *[discrimination]*<br><br>Did you feel happy with the care when you left / did you get what you wanted? *[needs met]*	

**Health seeking (HEALTH INFORMATION, ACCESS)**<br>**Discrimination/ culturally component care (ACCESS)**<br>**Barriers to and enablers of access (APPRAISE)**<br>**Enablers of care (UNDERSTANDING)**	Were there things that were good or done well (like what)? Things that you didn't like (like what)?<br>What changes do you think would make things better for your next visit to the doctor or nurse? *[solution focused]*	
**Enabling access - Unfamiliar with prevention (HEALTH INFORMATION)**	In Australia, women and girls are told about women's health checks at school and by doctors and nurses in health centres, how to find their way to health care and how to find information and women's health care.<br><br>What do you think are the best ways of telling newly arrived women about women's health checks? For example the 4 listed health problems above. What is the best way to get you the information you want about women's health? What else do you think would help?	E.G community groups, education sessions, magazines, websites, online videos

Before we finish, I would like to ask you if there is anything else about getting the health care, especially women's health care since you arrived in Australia, that you would like to discuss. Anything you would like to add?

Finishing

• Are there any other issues you would like to raise, or questions you have for me?

• Ask any demographic questions that have not already been covered and document on separate sheet (see below)

• Is there anyone else in your community you think might be interested in being interviewed?

Hand out information with details

• Would you like to receive an email with the results of this study? If yes, please spell out your email address, and I will add this to a confidential email list.

• Thank you so much for participating. We thank you all for your time and ideas. This has been extremely helpful. You have helped to provide a good understanding of the challenge's women face accessing care. Your contributions are greatly appreciated.

Interview Number: **Pseudonym:**

Sociodemographic data:

In which country were you born?

What year did you arrive in Australia?

Do you know what year you were born in or if you are not sure could you estimate it?

What is your marital status?

Do you have children? If yes, how many?

What kind of work do you do?

Are you working in paid employment?

What level of schooling have you completed, so far?
